# Transcriptome Profiling Reveals Features of Immune Response and Metabolism of Acutely Infected, Dead and Asymptomatic Infection of African Swine Fever Virus in Pigs

**DOI:** 10.3389/fimmu.2021.808545

**Published:** 2021-12-15

**Authors:** Hualin Sun, Qingli Niu, Jifei Yang, Yaru Zhao, Zhancheng Tian, Jie Fan, Zhonghui Zhang, Yiwang Wang, Shuxian Geng, Yulong Zhang, Guiquan Guan, David T. Williams, Jianxun Luo, Hong Yin, Zhijie Liu

**Affiliations:** ^1^African Swine Fever Regional Laboratory, China (Lanzhou) and State Key Laboratory of Veterinary Etiological Biology and Key Laboratory of Veterinary Parasitology of Gansu Province, Lanzhou Veterinary Research Institute, Chinese Academy of Agricultural Sciences, Lanzhou, China; ^2^Commonwealth Scientific and Industrial Research Organization (CSIRO) Australian Centre for Disease Preparedness, Geelong, VIC, Australia; ^3^Jiangsu Co-innovation Center for Prevention and Control of Important Animal Infectious Diseases and Zoonoses, Yangzhou, China

**Keywords:** African swine fever virus (ASFV), differential expression, functional analysis, immune response, metabolism, RNA-Seq

## Abstract

African swine fever virus (ASFV) infection can result in lethal disease in pigs. ASFV encodes 150-167 proteins, of which only approximately 50 encoded viral structure proteins are functionally known. ASFV also encodes some nonstructural proteins that are involved in the regulation of viral transcription, viral replication and evasion from host defense. However, the understanding of the molecular correlates of the severity of these infections is still limited. The purpose of this study was to compare host and viral gene expression differences and perform functional analysis in acutely infected, dead and cohabiting asymptomatic pigs infected with ASFV by using RNA-Seq technique; healthy pigs were used as controls. A total of 3,760 and 2,874 upregulated genes and 4,176 and 2,899 downregulated genes were found in healthy pigs vs. acutely infected, dead pigs or asymptomatic pigs, respectively. Additionally, 941 upregulated genes and 956 downregulated genes were identified in asymptomatic vs. acutely infected, dead pigs. Different alternative splicing (AS) events were also analyzed, as were gene chromosome locations, and protein-protein interaction (PPI) network prediction analysis was performed for significantly differentially expressed genes (DEGs). In addition, 30 DEGs were validated by RT-qPCR, and the results were consistent with the RNA-Seq results. We further analyzed the interaction between ASFV and its host at the molecular level and predicted the mechanisms responsible for asymptomatic pigs based on the selected DEGs. Interestingly, we found that some viral genes in cohabiting asymptomatic pigs might integrate into host genes (DP96R, I73R and L83L) or remain in the tissues of cohabiting asymptomatic pigs. In conclusion, the data obtained in the present study provide new evidence for further elucidating ASFV-host interactions and the ASFV infection mechanism and will facilitate the implementation of integrated strategies for controlling ASF spread.

## Introduction

African swine fever (ASF) is a highly contagious and fatal infectious disease affecting domestic pigs and wild boars. This disease is caused by African swine fever virus (ASFV). ASFV is a large double-stranded DNA (dsDNA) virus and is the only member of the genus *Asfivirus* in the family *Asfarviridae*. ASFV is a unique group of animal DNA arboviruses that depend on a complex transmission cycle involving soft ticks, sylvatic transmission and domestic pigs (1).

ASF was initially detected in Kenya, Africa, in 1921 (2). In 1957, the first transmission of ASFV outside of the African continent was observed, when it entered Europe *via* Portugal and then spread rapidly to Spain, France, Belgium, the Netherlands, and Italy (3). From 1971 to the 1980s, it was transferred from Spain to Cuba in South America. This wave of epidemics caused heavy economic losses in Cuba. Subsequently, ASF epidemics were reported in Brazil, Haiti and other American countries (4). In 2007, ASF spread from southeastern Africa to the Republic of Georgia (2, 5). In March 2017, an ASF epidemic occurred in Irkutsk, in an area relatively close to China (only approximately 1,000 km) (6). On August 3rd, 2018, an outbreak of ASF in pigs was first reported in Shenyang, Liaoning Province, China (7) and the virus shared 100% identity with the Georgian strain (Georgia 2007) found in Russia and other countries in Eastern Europe based on partial *B646L* gene sequence analysis (8). Soon, outbreaks of ASF were reported in Serbia, Slovakia (2019), Greece, Moldova and Germany (2020) (9).

ASFV has a linear dsDNA genome of 170 to 193 kb that contains 150 to 167 open reading frames (ORFs), encoding 150 to 200 proteins, among which approximately 54 proteins are structural proteins, while more than 100 nonstructural proteins have been identified. Some of these proteins have been found to be involved in virus and host interactions (10). Some were found to encode DNA replication, gene transcription and RNA modification, regulate host cell functions and participate in viral immune escape (11, 12). However, knowledge of the expression profile and the differences in host genes before and after ASFV infection is limited.

In this study, we analyzed the gene expression patterns of both hosts and viruses in spleens from infected pigs under different conditions on the basis of RNA-Seq. The common and unique gene expression patterns of acutely infected, dead, asymptomatic infected and clinically healthy pigs indicate the involvement of the modification of host immunity.

## Materials and Methods

### Facility and Ethics Statements

All samples were collected from pigs on a farm and subjected to laboratory tests at a biosafety laboratory-3 level (BSL-3) of the Lanzhou Veterinary Research Institute (LVRI), Chinese Academy of Agricultural Sciences (CAAS) accredited by the China National Accreditation Service for Conformity Assessment (CNAS) and approved by the Ministry of Agriculture and Rural Affairs. In the laboratory, to reduce any potential risk, protocols are strictly followed, all activities are monitored by professional staff of LVRI, and random inspections are conducted by the local and central governmental authorities without advance notice.

This study was approved by the Animal Ethics Committee of the Lanzhou Veterinary Research Institute, Chinese Academy of Agricultural Sciences. All animals were handled in accordance with the Animal Ethics Procedures and Guidelines of the People’s Republic of China.

### Field Samples Collection

An outbreak of ASF occurred on a swine farm in Qingyang City, Gansu Province, in 2019 (altitude: 885-2089 m). Clinical blood, heart, spleen, liver, lung, and kidney samples were collected from dead pigs that had been acutely infected with the disease and slaughtered cohabiting asymptomatic infected pigs (viral nucleic acid test-negative, antibody test-positive). Samples from healthy pigs were also collected from uninfected pigs that were free of other common viral diseases (such as foot-and-mouth disease virus (FMDV), porcine reproductive and respiratory syndrome (PRRSV) and classical swine fever virus (CSFV)) and were excluded by testing using commercial ELISA kits (ELISA Kit for Detecting Antibodies of FMDV produced by the LVRI of CAAS; IDEXX PRRS 3XR Ab ELISA and CSFV Ab ELISA kit purchased from IDEXX Laboratories, Inc., Westbrook, ME, USA) (data not shown). The samples positive for ASFV were evaluated *via* real-time PCR targeting the *B646L* (p72) gene and by ELISA. Spleens with a relatively high viral load from 3 ASFV-infected pigs and 3 cohabiting asymptomatic infected pigs were used for RNA-Seq analysis. Spleens from 3 healthy pigs were used as controls.

### Real-Time PCR

To quantify ASFV load in different samples, total genomic DNA was extracted from blood and different tissue homogenates using the QIAamp DNA blood Kit (QIAGEN, Maryland, USA) according to the manufacturer’s instructions. All prepared genomic DNA samples were stored at -20°C until use. qPCR was carried out on a CFX96 Touch Real-Time PCR instrument (Bio-Rad, USA) using Quick 96-Well Plates according to the OIE-recommended procedure described by King et al. (13).

### ELISA

To compare the differences in the ASFV antibody responses among acutely infected, dead, cohabiting asymptomatic and healthy pigs, two commercial kits (IDVET and INGEZIM PPA COMPAC R11.PPA. K3, INGENASA) were employed for the detection of antibodies, according to the manufacturer’s protocol. The test was considered valid when the OD value of the negative control (NC) was higher than 0.7 (OD _NC_ > 0.7) and when the OD _PC/_OD _NC_ was lower than 0.3 (OD _PC_/OD _NC_ < 0.3) with the IDVET kit. The S/N% of a sample was calculated as follows: S/N%= (OD _sample_ - OD_PC_)/(OD _NC_ - OD _PC_) ×100 (S/N% ≤ 40% positive, S/N% > 50% negative, between both values, doubtful). The test was considered valid when the OD value of the negative control (NC) was at least 4 times higher than that of the positive control (PC) with the INGENASA kit. The blocking rate of a sample was calculated as follows: blocking rate % = (OD _NC_ - OD _sample_)/(OD _NC_ - OD _PC_) ×100 (blocking rate ≥ 50% positive, blocking rate ≤ 40% negative, between both values, doubtful).

### Transcriptome Sequencing and Data Analysis

Total RNA extracted from each sample and an equal amount of total RNA from each group (Acutely infected, dead = 3, Cohabiting asymptomatic = 3, Healthy = 3) were used to construct libraries using the MGIEasy RNA Kit following the manufacturer’s instructions (BGI, Wuhan, China). Briefly, total RNA samples were digested with DNase I, followed by mRNA enrichment using oligo (dT) beads. The enriched mRNA was fragmented by using Tn5 transposase (BGE005, BGI), and cDNA was synthesized by adding random primers. The amplified cDNA was subjected to end repair and adaptor ligation and subjected to PCR. The PCR products were recovered and circularized to obtain a single-stranded circular DNA library. The final library was obtained after the digestion of uncircularized linear DNA molecules. The paired-end cDNA library was sequenced using the BGISEQ-500 system (BGI, Wuhan, China), and to obtain clean data, the filtering software SOAPnuke was used for statistics, Trimmomatic was used for filtering, and reads including adapters (adaptor contamination), reads with an unknown base (N) content greater than 5% and low-quality reads were removed. Thereafter, the RNA-Seq reads were employed for genome alignment using the Hierarchical Alignment for Spliced Transcripts (HISAT) program.

According to the HISAT2 results, the expression level of each gene was subsequently calculated as the fragments per kilobase of exon per million fragments (FPKM) value. Twofold variance in expression levels and *P* < 0.05 were used as the cutoffs to define differentially expressed genes (DEGs). P-values were calculated using R software (DESeq2) (14), where the DEseq2 method is based on the negative binomial distribution principle. We used the criteria of a fold difference | log2 (FC) | > 2 and corrected p ≤ 0.05 to screen for DEGs. Gene Ontology (GO) (15) analysis was performed using R software (cluster Profiler) to annotate molecular functions (MFs), cellular components (CCs) and biological processes (BPs), and Kyoto Encyclopedia of Genes and Genomes (KEGG) analysis was also conducted (16). All data were obtained from at least three replicates. ANOVA was performed using SPSS 12.0. Values of *P* ≤ 0.05 were assumed to be statistically significant.

### Statistical Analysis of Alternative Splicing Events

Alternative splicing (AS) events refer to a transcription process from a gene encoding mRNA to a mature mRNA that produces multiple different mature mRNAs through different cleavage modes and ultimately produces different proteins. We used rMATS software to analyze the differential splicing of genes on the basis of the results provided in transcript sequencing annotation files. We quantified the expression of alternative splicing events in different samples with the rMATS statistical model, calculated *P* values with the likelihood-ratio test to express the differences in inclusion levels (IncLevels) between the 3 groups of samples, and corrected the *P* values using the Benjamini Hochberg algorithm to obtain the Q values. There were five types of rMATS-identifiable alternative splicing events: skipped exons (SEs), alternative 5′ splice sites (A5SSs, or first-exon alternative splicing), alternative 3′ splice sites (A3SSs, or last-exon alternative splicing), mutually exclusive exons (MXE, or alternative exon skipping) and retained introns (RIs) (17).

### Protein-Protein Interaction Network Analysis

The obtained significant DEGs were analyzed online using STRING software (https://string-db.org). They were selected according to the criteria of a P value < 0.05 and | log2 (FC) | > 2 in differential expression gene screening. The possible interaction relationships of proteins encoded by the DEGs were then analyzed, and PPI interaction networks were constructed using Cytoscape Tool.

### Validation of Differentially Expressed Transcripts

To verify the high-throughput sequencing results from the RNA-Seq data, the upregulated or downregulated genes were detected by RT-qPCR. The primers used for qPCR are shown in **Table 1**. RNA samples were analyzed by one-step qPCR using a one-step PrimeScript RT-PCR kit (Perfect Real Time) according to the manufacturer’s protocol (Takara, Dalian, China). Quantitative real-time PCR assays were performed on a CFX96 Touch real-time PCR instrument (Bio-Rad, USA) using Fast 96-Well Plates containing 5 ng RNA per reaction in a reaction volume of 20 μL. Thermal cycling conditions were as follows: one cycle of 95°C for 30 s, 40 cycles of 95°C for 5 s and 55°C for 30 s, and one cycle of 95°C for 15 s, 55°C for 1 min and 95°C for 15 s. All samples were analyzed in triplicate. GAPDH was used as a reference housekeeping gene.

**Table 1:** Primers used in this study.

Targets	Sequences of primers (5’-3’)	References or GenBank Accession Numbers
ASFV-B646L	F:CTGCTCATGGTATCAATCTTATCGA	(13)
	P: FAM-CCACGGGAGGAATACCAACCCAGTG-TAMRA	
	R:GATACCACAAGATCRGCCGT	
ASFV-I73R	F:AGCACAATGTCGTCTTACCTACAGGA	MK333180.1
	R:TCCGTATCCAAAGCGGGGGA	
ASFV-DP96R	F:CTGAGAAGTCGGCCCGCGAA	MK333180.1
	R:TCTGGATGGAGCGCATTAGGGA	
ASFV-L83L	F:GCTGAGCCTGATAAAACAAACGA	MK333180.1
	R:TTTATGGCAACAATCTACCATTGAA	
Porcine-ELL3	F:CTGCTTCACCCCTGCTGCCC	XM_001928143.3
	R:TGTTGCCGCTGACACTCCTGC	
Porcine-ISG15	F:GGTGAAGATGCTGGGAGGCAAGG	NM_001128469.3
	R:TGCTGGAAGGCGGGCACAC	
Porcine-OASL	F:TCCTGCGACTGGTAAAACACTGGT	NM_001031790.1
	R:CGAGGGCATAGAGAGGGGGC	
Porcine-CD64	F:GCAGCCTCCGTGGGTCAGTG	KX890133.1
	R:CTGGGGGTCAAGGTCTCAATGGC	
Porcine-IRF7	F:CTGCCCCGAGACTGCGACAC	NM_001097428.1
	R:GGTCCTGCCCGAAGCCCAG	
Porcine-SAA3	F:AGCGATGCCAGAGAGAATGTCCAG	NM_001044552.1
	R:AAGTGGTTGGGGTCCTTGCCA	
Porcine-HBB	F:GCCCACGGCAAGAAGGTGC	NM_001144841.1
	R:GCGAGCCAGAACAACCACTATCAC	
Porcine-RSAD2	F:GCGGGCAGGGGGTGATAGGA	NM_213817.1
	R:TGGGGGTGGTGGGCAGATGG	
Porcine-DDX58	F:GCCACAACACCAGCAAACAGCA	NM_213804.2
	R:GCATCCCCAACGCCAACCGA	
Porcine-CD72	F:GCCTGCTCCTCACCTGCCTG	NM_001097493.1
	R:AATCTTCTTCCCTCTGCCCCAGC	
Porcine-HSH2D	F:CTGGGGCAGGCAACTCAGCC	NM_001243826.1
	R:GACCCTTGTGGTGGCCTCGC	
Porcine-UBE2J1	F:ACCCCTCAGCAGCATCCCCT	NM_001077219.1
	R:TCTTGGCTGCTGGCCCTGGA	
Porcine-TMPRSS2	F:TCAGTACCACCCGCCCTCGG	XM_021071009.1
	R:TCCATCTCGGGCGTGGAGCA	
Porcine-TLX1	F:GCACTGAGCGCTTCGGGTGT	XM_021073100.1
	R:CAGAGGGCACGGTGGGCAAG	
Porcine-TFR2	F:ACCCCGACGTCTACTGCCCC	XM_021086235.1
	R:CACTGGCCACCTTCTGGGCG	
Porcine-SH3GL3	F:CGGCTGCGTCCAATGTCCCC	XM_001929079.5
	R:CACGACAGCAGGGCTGGTCC	
Porcine-RAC3	F:TTGAGCGGCTGCGGGACAAG	XM_021066402.1
	R:CGGCTTCTTCACAGGGGGCG	
Porcine-PAX5	F:CGACTGCTTGCGGAACGGGT	XM_003122019.6
	R:GACACCTGCGTCACGGAGCC	
Porcine-MMP3	F:TGGGGTTGGAGGTGACGGGG	NM_001166308.1
	R:GGGCAGGCCTGGAAAGGTGC	
Porcine-JUN	F:GCAACAGCAACCACCGCAGC	NM_213880.1
	R:TCGATGGGAGACAGGGGCGG	
Porcine-IGFBP2	F:CAGCACCGGCAGATGGGCAA	HQ432890.1
	R:CACCAGCACTCCCCACGCTG	
Porcine-HFE	F:CGGGCTGCCCCTGTTTGAGG	XM_021098424.1
	R:TTACCCGAGAGCCAGGGGGC	
Porcine-FRMPD1	F:CCCCTGCGGACCACTTGCAG	XM_005660252.3
	R:GCACTGTCCGTCGTGCTGCT	
Porcine-CNFN	F:CAACCACCCGCAGCCCTCTG	XM_013988596.2
	R:GCAGCACTCGCCGAAGTCGT	
Porcine-CD79A	F:GCGCGTCCCTCAAGGCAACT	NM_001135962.1
	R:TGTCCAGGAAGGGCCTGGGG	
Porcine-CAV2	F:GACCGAGATCCCCACCGGCT	NM_001123091.1
	R:TTCCCGCAGCGAAGGCCAAG	
Porcine-BTK	F:TGCACCAAACAGCGCCCCAT	NM_001243576.1
	R:CGCCAGGTCTCGGTGGAGGA	
Porcine-BLNK	F:GCTGAGGAGGCCGGGGATGA	XM_001928233.5
	R:AGGGCGGAGACTGCCTCTGG	
Porcine-BAG2	F:TCAGCGCCAAGGCCAACGAG	XM_003128336.4
	R:CTGCCGCATGTCCTGGCTGT	
Porcine-AMPH	F:AGCGGGCTCTGCTGGAGTGA	NM_001244203.1
	R:TCCTCAGCCCGGGTGTCCAG	
Porcine-GAPDH	F:TGGAAAGGCCATCACCATCT	NM_001206359.1
	R:ATGGTCGTGAAGACACCAGT	

## Results

### Clinical Sample Analysis by qPCR and ELISA

**Figure 1:**
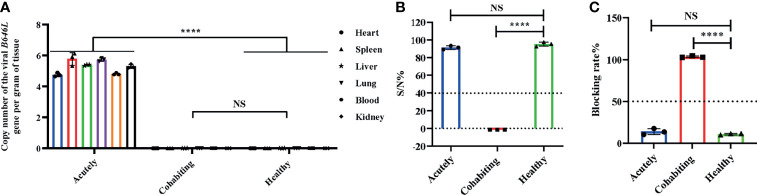
Clinical sample analysis by qPCR and ELISA. **(A)** ASFV *B646L* (p72) gene detection in different tissues by qPCR. DNA was extracted from blood, heart, spleen, liver, lung and kidney samples from acutely infected, cohabiting asymptomatic and healthy pigs. ASFV *B646L* gene copy numbers were quantified by qPCR assays, and the error bars represent the standard deviation among replicates. (Data are shown as the mean ± SD based on three independent experiments. **(B, C)** Analysis of ASFV antibody levels in serum samples from acutely infected, dead, cohabiting asymptomatic and healthy pigs. Sera were isolated from acutely infected, dead, cohabiting asymptomatic and healthy pigs. ASFV antibody levels were detected by using two commercial ELISA kits. The S/N% of a sample with the IDVET kit was calculated as follows: S/N%= (OD _sample_ - OD_PC_)/(OD _NC_ - OD _PC_) ×100 (S/N% ≤ 40% positive, S/N% > 50% negative, between both values, doubtful). The blocking rate of a sample with the INGENASA kit was calculated as follows: blocking rate % = (OD _NC_ - OD _sample_)/(OD _NC_ - OD _PC_) ×100 (blocking rate ≥ 50% positive, blocking rate ≤ 40% negative, between both values, doubtful). The error bars are the standard deviation among replicates. *P* < 0.0001 determined by two-tailed Student’ s t-test. (****P < 0.0001). NS, No significant.

To verify which clinical samples contained ASFV, qPCR and ELISA were performed. The PCR results showed that ASFV was detected in blood and tissue samples from acutely infected, dead pigs and was not found in samples from cohabiting asymptomatic and healthy pigs. The highest levels of viral DNA were found in the spleens of acutely infected pigs (**Figure 1A**). The antibody response results showed that the sera of cohabiting asymptomatic pigs were positive against ASFV P32 or VP72 protein (S/N% ≤ 40% or blocking rate ≥ 50%), while the sera from acutely infected, dead pigs and from healthy pigs were negative (S/N% > 50% or blocking rate ≤ 40%) (**Figures 1B, C**).

### Transcriptome Analysis and Gene Expression Statistics

**Figure 2:**
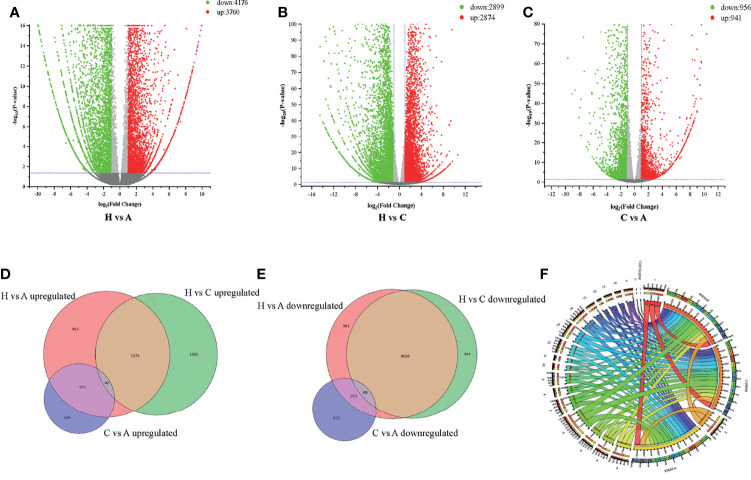
Differentially expressed genes (DEGs) analysis. **(A)** Healthy vs. acutely infected, dead pig volcano plot. **(B)** Healthy vs. cohabiting asymptomatic pig volcano plot. **(C)** Cohabiting asymptomatic, dead vs. acutely infected pig volcano plot. The red dots correspond to upregulated genes, the green dots correspond to downregulated genes, and the gray dots represent the genes without statistically significant differences. **(D)** Venn diagram of significantly upregulated genes in healthy vs. cohabiting asymptomatic pigs; **(E)** Healthy vs. cohabiting asymptomatic pigs; and acutely infected, dead vs. cohabiting asymptomatic pigs. The intersection of the circles represents genes that are significantly up- or downregulated by different partial mechanisms at the same time. **(F)** Significant DEGs mapped in chromosome Circos plots. 1-18 and XY represent the pig chromosomes, and MK33180.1 represents the ASFV gene set. Bands of different colors represent the numbers of genes identified by RNA-Seq in different groups to be mapped on chromosomes.

**Figure 3:**
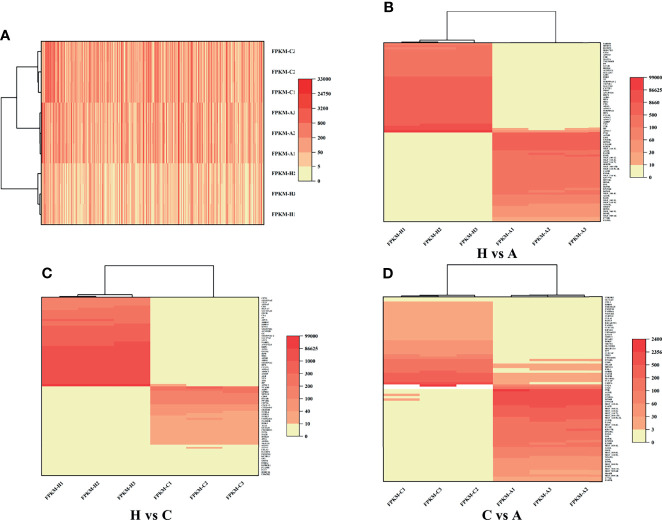
Heatmap of DEGs. **(A)** DEGs identified in healthy, cohabiting asymptomatic and acutely infected, dead pigs by RNA-Seq. The top 500 significant DEGs from both virus and host with the smallest *P* value were selected. **(B)** Significant DEGs identified in healthy vs. acutely infected, dead pigs. **(C)** Significant DEGs identified in healthy vs. cohabiting asymptomatic pigs. **(D)** Significant DEGs identified in cohabiting asymptomatic vs. acutely infected, dead pigs.

RNA-Seq is a very powerful technique and has been widely used in the fields of oncology, immunology and cell biology in recent years; it is also used in the ASFV mechanisms and regulation of ASFV transcription (18–21). To investigate the differential gene expression between acutely infected, dead, cohabiting asymptomatic and healthy pigs, we compared the gene expression at the transcriptional level by RNA-Seq technique. Each of nine samples tested in the BGISEQ500 platform produced an average of 6.42 GB of data. As a result, the average ratio of samples to the *Sus scrofa* reference genome (Organism name: *Sus scrofa* (pig), Source: NCBI_Sscrofa11.1 and ASFV-MK333180, Reference Genomic Version: https://www.ncbi.nlm.nih.gov/assembly/GCF_000003025.6/ and https://www.ncbi.nlm.nih.gov/genome/?term=MK333180) was 84.13%, and the average ratio to gene sets was 29.57%. The number of predicted novel genes was 4673; the total number of detected expressed genes was 22,886, including 18,231 known genes and 4,655 predicted novel genes. A total of 45,891 novel transcripts were detected, of which 15,759 were novel alternatively spliced isoforms of known protein-coding genes and 4,673 were transcripts of novel protein-coding genes. The remaining 25,459 were long noncoding RNAs. Expression level differences | log2 (FC) | > 2 were considered to indicate significant differential expression in healthy vs. acutely infected, dead pigs; healthy vs. cohabiting asymptomatic pigs; or cohabiting asymptomatic vs. acutely infected, dead pigs. The comparisons of these groups revealed 3,760, 2,874 and 941 upregulated genes and 4,176, 2,899 and 956 downregulated genes, respectively (**Figures 2A–C**). Furthermore, 40 genes were significantly upregulated and 68 genes were significantly downregulated in all 3 comparison groups, and the number of simultaneously downregulated genes in the acutely infected, dead pigs and cohabiting asymptomatic pigs was not significant (**Figures 2D, E**). Chromosomal mapping of significant DEGs was also performed (**Figure 2F**). In addition, the top 500 significant DEGs with the smallest *P* value were selected and analyzed for expression of both ASFV and host genes among 3 groups (**Figure 3A**). We further screened the top 40 significantly upregulated and significantly downregulated genes in each group for heatmap (**Figures 3B–D**). ASFV genes were mainly upregulated in acutely infected, dead pigs; these genes included important virulence genes and multigene family genes (MGF-110-1L; MGF-360-15R; MGF-110-9L) (**Figure 3B**), the major genes upregulated in cohabiting asymptomatic pigs were derived from the host (**Figure 3C**). Furthermore, a small number of viral genes were detected in cohabiting asymptomatic pigs. However, the major genes encoding ASFV structural proteins and important virulence genes were not detected in cohabiting asymptomatic pigs (**Figure 3D**). The RNA-Seq data have been successfully deposited in the SRA database (https://www.ncbi.nlm.nih.gov/sra/PRJNA778812) with accession number PRJNA778812.

### GO Analysis of DEGs

**Figure 4:**
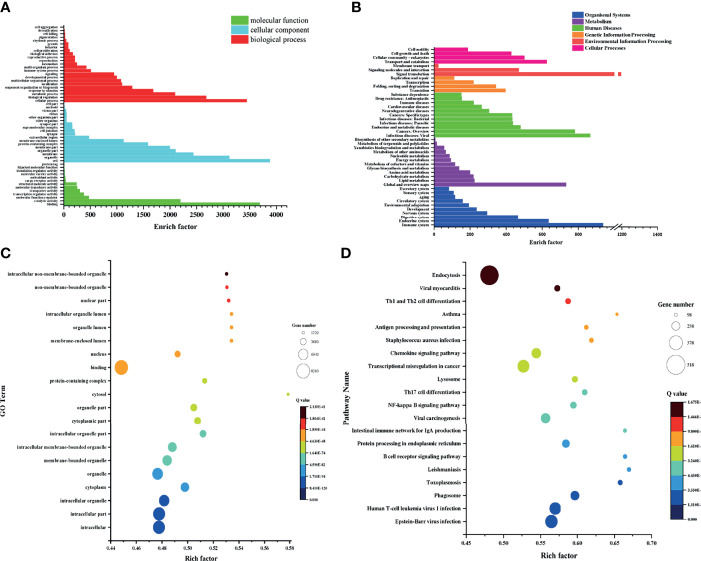
GO and KEGG analysis and enrichment of genes identified in each group. **(A)** GO analysis of cohabiting asymptomatic vs. acutely infected, dead pigs. **(B)** KEGG analysis of cohabiting asymptomatic vs. acutely infected, dead pigs. **(C)** Bubble diagram of GO enrichment in cohabiting asymptomatic vs. acutely infected, dead pigs. **(D)** Bubble diagram of KEGG enrichment in cohabiting asymptomatic vs. acutely infected, dead pigs.

To analyze the potential biological functions of the DEGs identified in the 3 groups, we performed GO annotation of the identified DEGs, in which three different categories, MF, CC and BP, were considered. In this study, the identified MF terms mainly included binding, catalytic activity, molecular function modulator and transcriptional regulator activity; CC terms mainly included cell, organelle, membrane and organelle parts; and BP terms mainly included cellular process, biological regulation, metabolic process and response to stimulation. The GO analysis and enrichment results of the transcripts from the 3 groups were similar (**Figures 4A, C**) (**Supplementary Figures S1** **A**–**D**).

### KEGG Analysis of DEGs

To further understand the biological functions in which the DEGs were involved, we conducted searches of the functional enrichment of the DEGs in the KEGG database. The results showed that the DEGs were mostly related to the immune system, endocrine system, metabolism, carbohydrate metabolism, viral infectious diseases, endocrine and metabolic diseases, transcription, replication and repair and signal transduction, and the KEGG analysis results of the 3 groups were similar. The top 20 pathways with the smallest Q values were selected for KEGG enrichment analysis. The DEGs identified in healthy pigs vs. acutely infected, dead pigs were mainly involved in the spliceosome, the B cell receptor signaling pathway, protein processing in endoplasmic reticulum, endocytosis, viral carcinogenesis, and the cell cycle. The DEGs identified in healthy pigs vs. cohabiting asymptomatic pigs were mainly involved in protein processing, endocytosis, the B cell receptor signaling pathway, the MAPK signaling pathway, transcriptional dysregulation in cancer, the spliceosome, the NF-κB signaling pathway, and FcγR-mediated phagocytosis in the endoplasmic reticulum (**Supplementary Figures S2A**–**D**). The DEGs identified in cohabiting asymptomatic vs. acutely infected, dead pigs were mainly involved in the phagosome, the B cell receptor signaling pathway, protein processing in the endoplasmic reticulum, the intestinal immune network produced by IgA, viral carcinogenesis, the NF-κB signaling pathway, and Th17 cell differentiation (**Figures 4B, D**). We compared the four pathways that were coenriched among the groups and compared the changes in significant DEGs among them (**Table 2**). In the B cell receptor signaling pathway, BLNK, BTK, CD96A, SASH3, MAB21L3, JUN, RAC3, and FOS were significantly upregulated and downregulated in acutely infected, dead pigs and cohabiting asymptomatic pigs. The differential expression of these genes may be related to the B cell response against infection. In the endocytosis pathway, HSH2D, AMPH, STARD7SNX32, TFR2, AZGP1, ROGDI, FGFRL1, FGFR3, PYGO1, and ESPN were significantly upregulated or downregulated in acutely infected, dead pigs and cohabiting asymptomatic pigs. In addition, our results showed that HSH2D was upregulated in cohabiting asymptomatic pigs but with a lower expression level than in acutely infected, dead pigs. These results suggest that viral invasion of the host causes significant differential expression of a large number of host genes involved in multiple signaling pathways, and most of these DEGs are related to host resistance to the virus as well as tissue damage repair.

**Table 2:** Different groups of differently expressed genes involved in KEGG.

Healthy vs. acutely infected, dead pigs								
B cell receptor signaling pathway	log2 (Fold Change)		Endocytosis	log2 (Fold Change)	Transcriptional misregulation in cancer	log2 (Fold Change)	Protein processing in endoplasmic reticulum	log2 (Fold Change)
**Significantly upregulated genes**								
BLNK	5.94		HSH2D	11.11	MMP3	9.48	UBE2J1	4.59
CD79A	5.66		AMPH	7.36	TLX1	8.54	BAG2	3.68
RASGEF1A	5.03		SH3GL3	5.06	A179L	6.97	HSPA14	3.56
RASGRP3	4.05		STARD7	4.85	WT1	5.08	AMBN	3.55
BTK	3.97		SH3KBP1	4.627	IL6	5.07	XAF1	3.49
RAC2	3.87		DEPDC1B	3.99	ARNT2	4.72	TICRR	3.41
STAC2	3.80		SNX32	3.93	AZU1	4.17	EIF2S1	3.06
SASH3	3.59		ARPC5L	3.85	CCND2	3.69	HSPA2	2.98
NRAS	3.33		STAC2	3.80	BCL2A1	3.39	BAK1	2.94
CARD11	3.27		POF1B	3.66	TAF15	3.27	ANKRD33B	2.59
**Significantly downregulated genes**								
MAB21L3	-4.97		TFR2	-10.26	IGFBP1	-11.67	C9H1orf116	-8.03
JUN	-4.86		AZGP1	-9.72	IGFBP2	-10.98	ESPN	-5.62
RAC3	-4.15		ROGDI	-9.61	TMPRSS2	-6.90	RRBP1	-5.16
FOS	-4.07		FGFRL1	-8.85	C15H2orf72	-6.61	SVIP	-5.01
YPEL2	-2.41		FGFR3	-8.77	IGF1	-5.74	MAN1C1	-4.99
			PYGO1	-6.19	CD14	-5.69	PRADC1	-4.39
			FXYD7	-5.98	SPINT1	-5.65	TMEM161A	-4.15
			ESPN	-5.62	GADD45G	-5.64	GKAP1	-4.14
			FOLR2	-5.41	GOLIM4	-5.54	C2H19orf24	-3.96
			LMF1	-4.91	TSPAN7	-5.48	CANX	-3.83
**Healthy vs. cohabiting asymptomatic pigs**								
**B cell receptor signaling pathway**	**log2 (Fold Change)**	**Endocytosis**		**log2 (Fold Change)**	**Transcriptional misregulation in cancer**	**log2 (Fold Change)**	**Protein processing in endoplasmic reticulum**	**log2 (Fold Change)**
**Significantly upregulated genes**								
CD79A	9.67	HSH2D		8.06	TLX1	9.12	BAG2	4.05
CD19	6.45	AMPH		7.11	FRMPD1	7.75	CNFN	3.66
CR1	6.31	GSG1L		6.00	PPARG	7.68	UBE2J1	3.65
CD22	5.61	CXCR2		5.04	GSG1L	6.00	HSPA2	3.32
BLNK	5.41	STARD7		4.92	MMP3	5.72	ARID5A	3.04
SASH3	4.84	MAATS1		4.71	WT1	5.70	HSPA14	3.01
CD79B	4.78	CXCR4		4.59	PAX5	5.26	TICRR	2.98
BTK	4.62	SNX32		4.51	ARNT2	5.22	EIF2S1	2.73
GRAP	4.49	STAC3		4.49	LMO1	4.34	ERO1A	2.73
INPP5D	4.45	SH3GL3		4.45	CD40	3.97	BAX	2.71
**Significantly downregulated genes**								
FOS	-4.44	AZGP1		-12.23	IGFBP1	-12.09	C9H1orf116	-8.16
JUN	-4.10	TFR2		-8.60	IGFBP2	-10.12	ANKRD33	-6.37
MAB21L3	-3.28	FGFRL1		-8.21	TMPRSS2	-7.93	LMAN1	-5.46
KRAS	-2.63	FGFR3		-7.61	NUPR1	-6.56	RRBP1	-5.45
		CMTM8		-5.56	MET	-5.95	SVIP	-5.31
		ESPN		-4.80	GOLIM4	-5.90	C2H19orf24	-5.10
		WASL		-4.73	ANKDD1B	-5.75	ESPN	-4.79
		PYGO1		-4.71	IGF1	-5.74	PRADC1	-4.54
		ROGDI		-4.60	GADD45G	-5.00	SEC62	-4.25
		LMF1		-4.58	CD14	-4.55	MAN1C1	-4.12
**Cohabiting asymptomatic vs. acutely infected, dead pigs**								
**B cell receptor signaling pathway**	**log2 (Fold Change)**		**Endocytosis**	**log2 (Fold Change)**	**Transcriptional misregulation in cancer**	**log2 (Fold Change)**	**Protein processing in endoplasmic reticulum**	**log2 (Fold Change)**
**Significantly upregulated genes**								
			XAF1	4.07	A179L	6.58	XAF1	4.07
			HSH2D	3.56	FCGR1A	5.27	TXNDC5	2.34
			POF1B	3.02	NUPR1	4.72	MFSD2B	2.24
			CAV2	2.43	MMP3	4.42	LMAN1	2.19
			CAV1	2.17	IFI6	4.36	ERN1	2.03
			FAM174B	2.12	ISG12(A)	4.22	SEC24A	2.02
					GZMB	3.38	SEC24D	2.01
					IL6	2.60		
					CXCL8	2.39		
					AZU1	2.37		
**Significantly downregulated genes**								
CR1	-4.97		GSG1L	-4.60	FRMPD1	-6.29	OS9	-2.89
CD79A	-4.06		CXCR2	-4.45	CSF1R	-5.38	CENPV	-2.87
CD19	-3.77		FOLR1	-3.70	PPARG	-5.19	CNFN	-2.41
CD22	-3.13		HFE	-3.42	GSG1L	-4.60	PHLDB3	-2.11
CD72	-3.10		DNM1	-2.78	SLC45A3	-4.10		
CD79B	-2.72		CCER2	-2.75	ETV5	-3.93		
RAC3	-2.14		FXYD7	-2.52	PAX5	-3.39		
YPEL2	-2.13		TNFAIP8L2	-2.49	CEBPA	-3.1		
			PRKCZ	-2.28	SPINT1	-3.10		
			GRK3	-2.24	TRABD2A	-2.92		

### Statistical Analysis of Alternative Splicing Events

**Figure 5:**
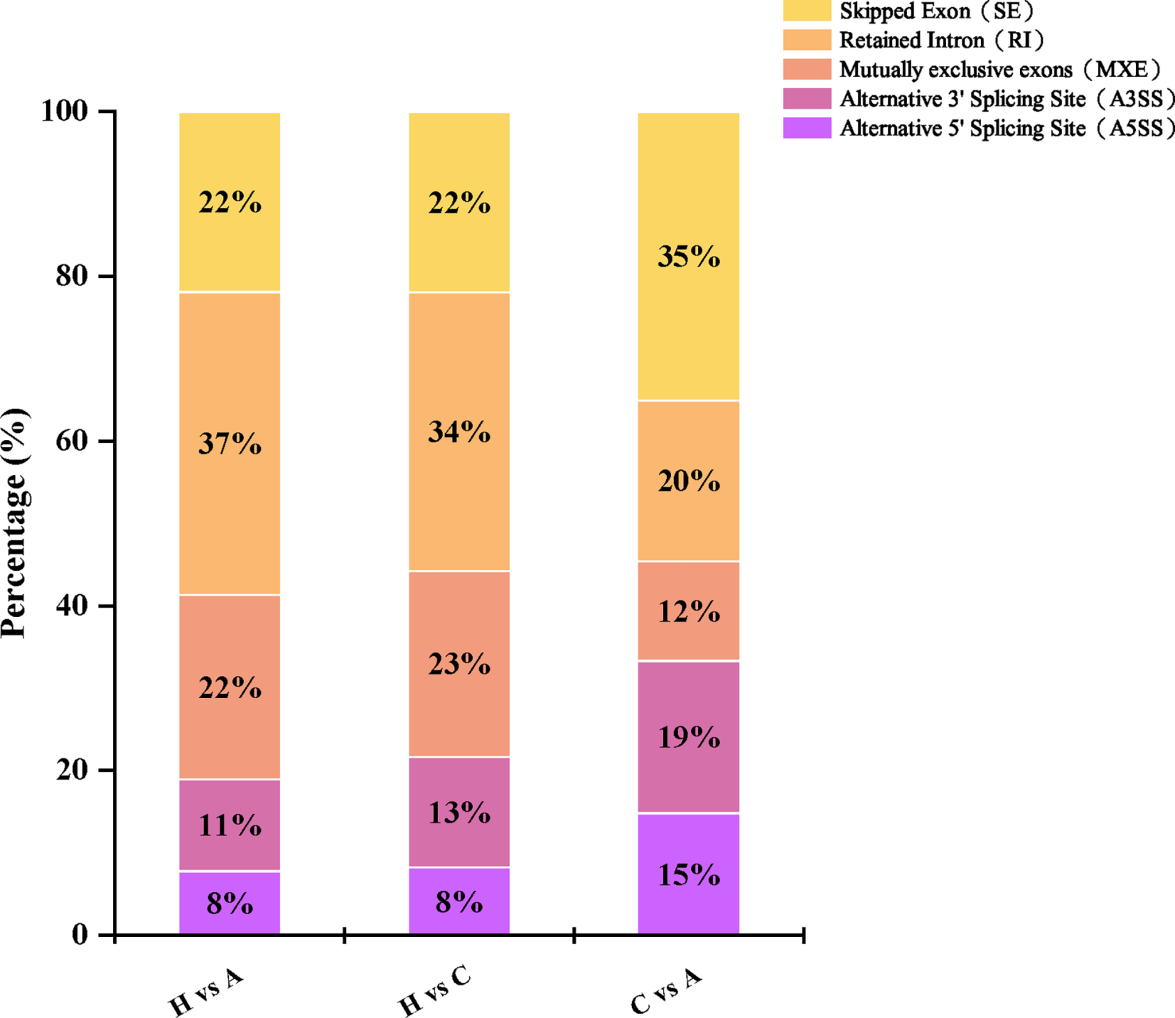
Variable splicing events among genes identified by RNA-Seq in different groups.

Among the different types of variable splicing events detected, the numbers of RI, SE, MXE, A5SS, and A3SS events were 891, 649, 569, 362, and 246, representing 32.8%, 23.9%, 20.9%, 13.3%, and 9.1%, respectively. Among the different groups of variable splicing events, healthy vs. cohabiting asymptomatic pigs showed the highest number of variable splicing events (1,298), followed by healthy pigs vs. acutely infected, dead pigs (1,014). The variable splicing events differed in cohabiting asymptomatic vs. acutely infected, dead pigs (405), where SE was the most frequent type of event observed, followed by RI, A3SS, A5SS, and MXE (142, 79, 75, 60, and 49 events, respectively) (**Figure 5**).

### PPI Analysis

**Figure 6:**
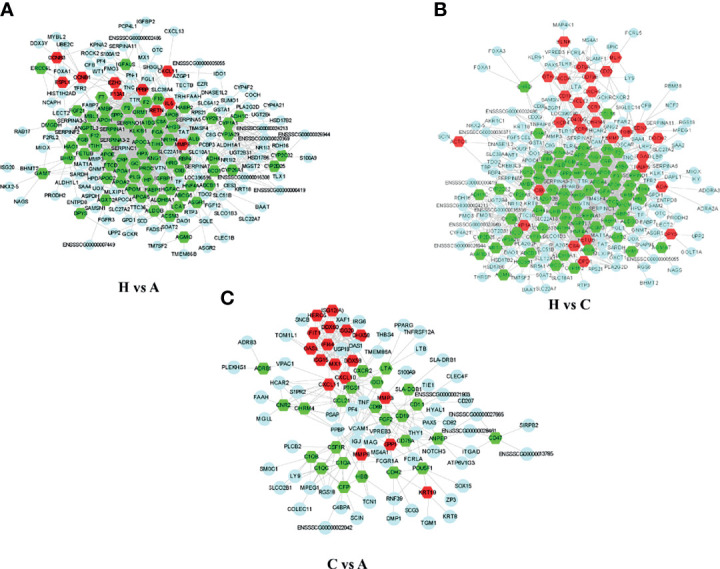
Network diagram of PPIs among the significantly DEGs in each group. **(A)** Significant DEG PPI network diagram of healthy vs. acutely infected, dead pigs. **(B)** Significant DEG PPI network diagram of healthy vs. cohabiting asymptomatic pigs. **(C)** Significant DEG PPI network diagram of cohabiting asymptomatic vs. acutely infected, dead pigs. The red pattern represents significant upregulation. The green pattern represents significant downregulation. The blue pattern represents interacting genes.

To predict the potential functions of the DEGs based on transcript analysis, we performed an online analysis of the significant DEGs obtained using STRING software (https://tring-db.org) according to the criterion of *P* value < 0.05, | log2 (FC) | > 2, and an interprotein interaction score > 0.4 for core DEG screening. We selected the top 250 significantly downregulated and upregulated genes identified in healthy vs. acutely infected, dead pigs; healthy vs. cohabiting asymptomatic pigs; and cohabiting asymptomatic vs. acutely infected, dead pigs for protein interaction prediction analysis, and we constructed a protein interaction network with the Cytoscape Tool. A total of 10, 29, and 17 core genes were significantly upregulated, and 75, 74, and 24 core genes were significantly downregulated in healthy vs. acutely infected, dead pigs; healthy pigs vs. cohabiting asymptomatic pigs; and cohabiting asymptomatic vs. acutely infected, dead pigs, respectively. The proteins expressed from these genes, such as ISG15, HBB, OASL, ITGB2, and IL6, were shown to have antiviral or immunomodulatory effects on other viruses (**Figures 6A–C**) (22–26).

### Validation of Differentially Expressed Transcripts by RT-qPCR

**Figure 7:**
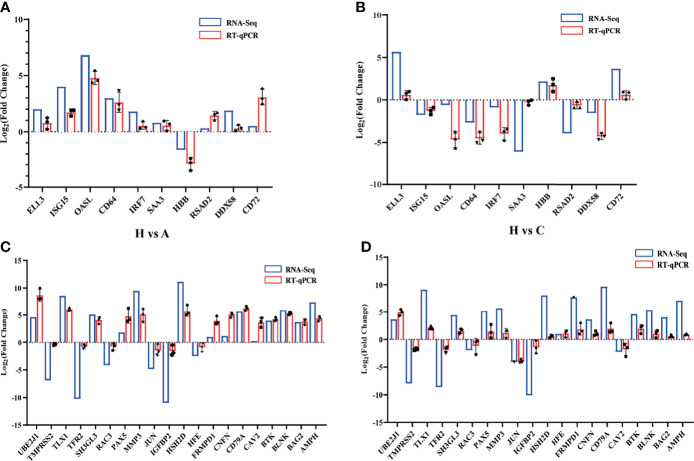
Validation of randomly selected DEGs. **(A)** Validation of 10 significant DEGs between healthy and acutely infected, dead pigs (H vs. A) by qPCR. **(B)** Validation of 10 significant DEGs between healthy and cohabiting asymptomatic pigs (H vs. C) by RT-qPCR using the log_2_ (fold Change) method. **(C)** Validation of KEGG pathway enrichment 20 genes between healthy and acutely infected, dead pigs (H vs. A) by qPCR. **(D)** Validation of KEGG pathway enrichment 20 genes between healthy and cohabiting asymptomatic pigs (H vs. C) by RT-qPCR using the log_2_ (fold Change) method. The RNA expression of each target gene was normalized to GAPDH expression. Three biological replicates were set up for each sample.

To validate the high-throughput sequencing results, significant DEGs were detected by RT-qPCR. ISG15, OASL, CD64, IRF7, SAA3, RSAD2, and DDX58, were significantly upregulated in healthy vs. acutely infected, dead pigs but downregulated in healthy vs. cohabiting asymptomatic pigs. HBB was significantly downregulated in healthy vs. acutely infected, dead pigs but significantly upregulated in healthy vs. cohabiting asymptomatic pigs. ELL3 and CD72 were upregulated in the two groups (healthy vs. acutely infected, dead pigs and healthy vs. cohabiting asymptomatic pigs) of samples (**Figures 7A, B**). Furthermore, some genes enriched in the KEGG pathway were detected by RT-qPCR, and the results were consistent with those in RNA-Seq (**Figures 7C, D**).

## Discussion

The virulence of ASFV strains ranges from highly pathogenic, causing death within a few days, to weakly pathogenic, causing subclinical or persistent infections with low levels of morbidity and mortality. The degree of ASF is related to viral and host factors (27). The virulence of ASFV in countries outside of Africa has gradually weakened, causing chronic infections in domestic pigs. It is clear that ASFV is evolving toward causing recessive infection in pigs (28). The highly pathogenic strain of ASFV mainly infects the mononuclear macrophage system, which can cause severe tissue necrosis, hemorrhage and eventually death. Moderately virulent ASFV also infects mononuclear macrophages, but the degree of disease is relatively mild. The replication ability of ASFV and the ability to cause macrophage lesions are the main factors used to measure the virulence of a virus (27). In China, ASFV has been reported to belong to genotype II, which is a high-virulence strain. In our study, ASFV was detected in blood and tissues from acutely infected, dead pigs and was not detected in cohabiting asymptomatic and healthy pigs by PCR. In contrast, an ASFV-specific antibody response was not detected in the sera of acutely infected, dead pigs or clinically healthy pigs but was detected in cohabiting asymptomatic pigs using ELISA. This indicated that in the same flock of pigs, some pigs might survive even though most of them suffered acute forms and died.

It is well known that RNA-Seq is an effective technique to address multiple clinical questions. Previous studies on ASFV transcriptome were based on the *in vitro* cell culture or experimental ASFV infection of animals and mainly analyzed for ASFV DEGs, but not paid close attention to host DEGs (18–21). In this study, the samples for RNA-Seq were collected from naturally infected animals in a farm, thus more relevant information and DEGs for both virus and host from animal samples could be obtained which truly reflected the animals infected status. Some differences in gene expression and biological processes were identified, mainly between spleen samples from acutely infected, dead pigs and cohabiting asymptomatic pigs. The significant DEGs identified in the spleen samples of acutely infected, dead pigs were mostly related to viral resistance, the stimulation of the macrophage antiviral response, the inflammatory response and the inhibition of viral replication (including genes such as OASL, ISG15, TNF, FCGR1A, and Mx1) (22, 24, 29–31), as well as cytoskeletal involvement and GTPase activity (including genes such as MASTL, GVIN1, and RHPN2). DEGs such as IDO1, DDX58, and IFIT3 were related to NF-κB pathway activation driven by IFN-γ and IFN-β (32). Genes implicated in the regulation of immune activity (including genes such as NPG1, were also identified). Most of the significant DEGs identified in spleen samples from cohabiting surviving pigs were related to the cytoskeleton, tissue damage repair, the cell cycle, immune regulation and cell replication (including genes such as BMP7, TGFBI, SPIB, CD68, SPIC, ELL3, and EIF1AY) (32–37). The significant DEGs encoding transmembrane proteins and receptor proteins included FCAMR, TMEM125, LTB4R2, PLVAP, and CSF1R (38–40). These results suggest that after viral invasion of the host, it causes significant differential expression of a large number of genes in multiple signaling pathways of host genes, and most of these differentially expressed genes are related to host resistance to the virus as well as tissue damage repair (35).

**Figure 8:**
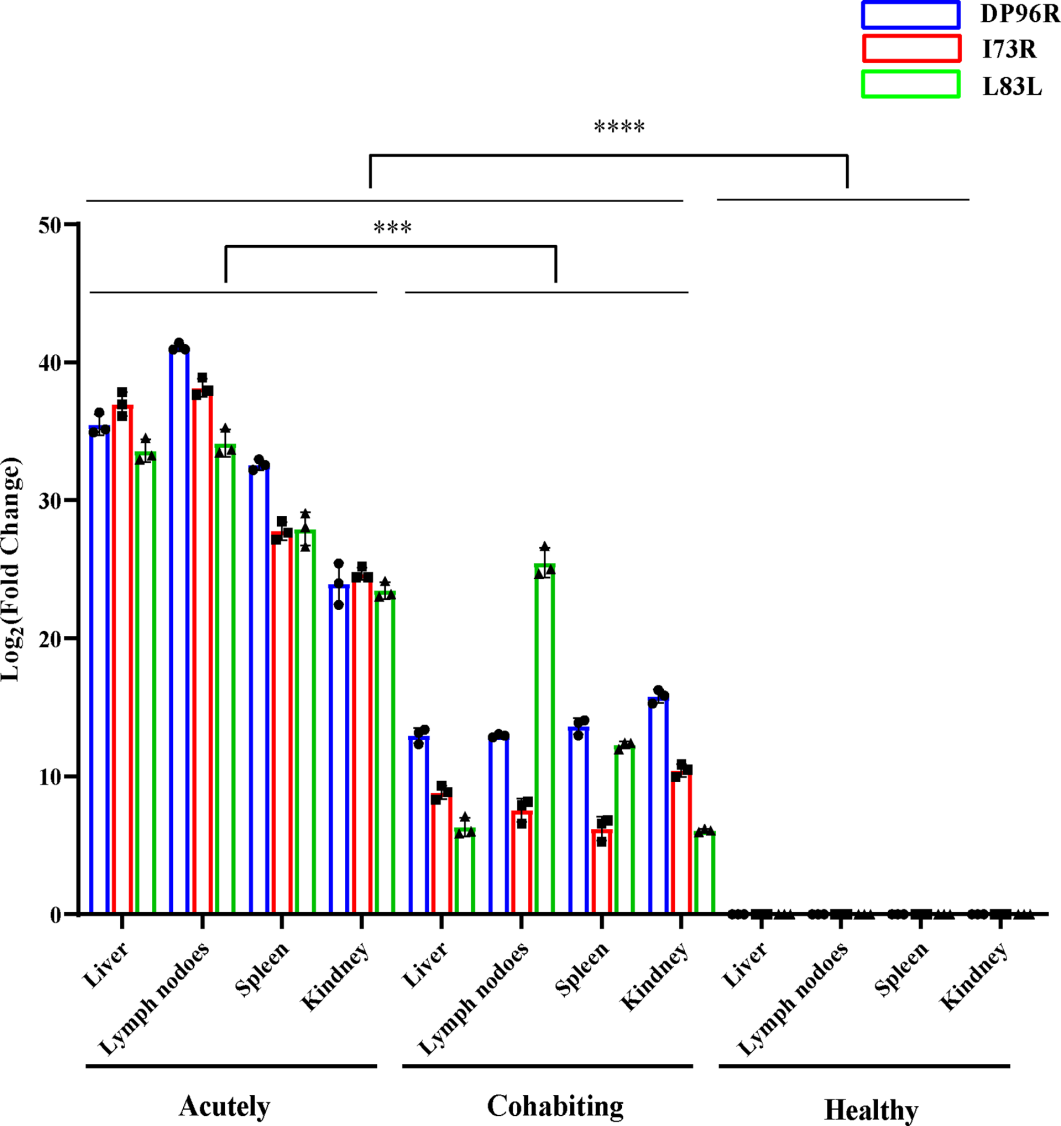
PCR confirmation of ASFV L83L, DP96R and I73R gene expression. The gene expression levels of L83L, DP96R and I73R in different tissues were detected by Q-PCR. The RNA expression level of each target was normalized to GAPDH. Error bars show the SD of replicate qPCR experiments. All experiments were independently conducted at least 3 times. Statistical significance is denoted by ***P < 0.001, ****P < 0.0001.

It is worth noting that ASFV gene transcripts were not completely absent from the tissue samples of cohabiting asymptomatic pigs, in which proteins such as L83L, I73R and DP96R were detected. L83L and I73R are genes expressed in the early transcriptional stage of ASFV; in particular, L83L is a transiently expressed early viral protein encoded by a nonessential gene that can bind to IL-1β and may be associated with the regulation of inflammatory cytokine activity (41). In a previous study, the DP96R protein was shown to act as a potential immune escape protein that can inhibit cGAS/STING-mediated IFN-β and NF-κB activation, the phosphorylation of TBK1 and the TBK1-mediated antiviral response (42). *B646L* (p72), *E183L* (p54), *CP204L* (p30), *EP402R* (CD2v) and other structural proteins have been reported to show important functions in virus attachment, invasion and host immune response (43–45), but these genes were not detected in cohabiting asymptomatic pigs through RNA-Seq analysis. However, some ASFV genes (L83L, I73R and DP96R) were still expressed in cohabiting asymptomatic pigs. One possibility is that the genes encoding these viral genes may be partially integrated into the host genome and transcribed with the host’s genes. It is well-known that retrovirus sequences are present in animal genomes, and these viruses integrate into the chromosomal DNA of the host as part of their normal replication cycle. However, in recent years, a novel way of analyzing virus evolution, which is the analysis of endogenous viral elements (EVEs)—ancient viral sequences integrated into the host genome revealed that a large and diverse population of sequences derived from nonretroviral viruses in animal genomes were identified and maintained in the host genome over many millions of year**s** (46). In a previous study, it was proven that ASFV and its hosts have undergone a long time of coevolution, and endogenous viral elements of ASFV might have been integrated into transmitted vector soft tick genomes (47). However, how did the viral genes integrate into the host genome? In a previous study, HBV DNA integration into the host genome occurred throughout the host genome at dsDNA breaks or was associated with genomic and chromosomal instability (48). Whether ASFV gene integration into the host genome has a similar mechanism remains to be proven. Another possibility is that some viral gene fragments remained in the tissues of cohabiting asymptomatic pigs and were not thoroughly cleaned by the host immune system. In addition, the relative expression level of these viral genes was obviously higher than that of other viral genes in acutely infected, dead pigs, and it might be that the elimination of these viral genes was slow in the host. Furthermore, consistent with the phenomenon observed in RNA-Seq analysis, the results of RT-qPCR indicated that these genes (L83L, I73R and DP96R) were successfully detected in tissues of the cohabiting asymptomatic pigs (**Figure 8**). Hence, their products could be used as specific markers for identifying pigs surviving from ASFV infection. The virus may recover its virulence under these conditions once stimulated by another virus or external stimuli.

After infection of the host, ASFV enters the cytoplasm through the endosomal pathway, *via* dynamic protein- and clathrin-mediated cholesterol-dependent endocytosis and giant cell proliferation. Then, it forms viral factories (VFs) around the nucleus (49), resulting in the accumulation of viral DNA and proteins in a microtubule-dependent manner, while ASFV envelope synthesis occurs in the host endoplasmic reticulum. Finally, the assembled virus is released into the extracellular space by budding (50). Cuesta-Geijo et al. showed that transport through the capsule and macrophage pinocytosis and GTPase activity are important pathways and factors in ASFV infection (51). PAK1, a kinase associated with several viral entry processes that can promote ASFV entry, was upregulated in acutely infected, dead pigs according to our RNA-Seq results, consistent with a previous report (52). The response to type I interferon (IFN-I) is suppressed upon virulent ASFV infection (53). The JAK-STAT pathway is activated by secreted IFNα1 (α/β), and STAT 1 and 2 are phosphorylated, leading to dimerization and binding to IRF9 to form an interferon-stimulated gene factor 3 (ISGF3) complex. ISGF3 can translocate to the nucleus and activate the transcription of several hundred interferon-stimulated genes (ISGs) (54). Some interferon genes show anti-viral activity. According to our results, interferon genes such as ISG15, ISG20, and ISG12 (A) were significantly upregulated in acutely infected, dead pigs and may show relationships with the host response to the virus through the IFN-I pathway. In the early stages of ASFV infection, the production of C-type lectin domain proteins is induced to inhibit caspase-3 activation through the p53 pathway and inhibit apoptosis to maintain the viral replication environment, while in the later stages of infection, these proteins promote apoptosis, release virions, and promote viral spreading (55). Some C-type lectin transcript variants belonging to different domain families, such as CLEC4E, CLEC1A, and CLEC1B, were observed to be significantly upregulated in acutely infected, dead pigs. These genes may be associated with virus-induced host resistance to apoptosis. In addition, we observed that caspase-3 was significantly upregulated. These results may indicate that there are effects both inhibiting and promoting apoptosis in host cells during viral infection at different stages, and such a mechanism would contribute to viral transcription, replication, and release.

We observed that CD68, GPR82, CD79α, ITGAD, AQP3, DOK3, CXCL13, and JPT2 were significantly upregulated in cohabiting asymptomatic pigs; these proteins are involved in immune regulation and cell proliferation. CD79α plays an important role in B cell development or the activation of downstream signals (56). AQP3 has a significant effect on the adipogenic differentiation and proliferation of intramuscular adipocytes (57). JPT2 is a microtubule-associated protein homolog that may have some effects on ASFV DNA and protein accumulation (50). It was also observed that proteins such as GC, CCL16, HPX, RBP4, C8B, and GSTA1 were significantly downregulated in cohabiting asymptomatic pigs; these proteins are involved in circulatory metabolism and immune regulation pathways (58). These significantly differentially expressed molecules may play important roles in opposing ASFV, but they may also be associated with resistance in cohabiting asymptomatic pigs, which needs to be further studied.

In conclusion, the results of viral and host gene expression analysis during ASFV infection were consistent with previous data describing host responses to ASFV infection (21, 59). Our study provides new knowledge regarding ASFV-infected hosts. This information will be useful for developing vaccines and small molecule compounds that will protect against ASF and for investigating the pathogenic mechanism of this disease.

## Data Availability Statement

The original contributions presented in the study are publicly available. This data can be found here: https://www.ncbi.nlm.nih.gov/bioproject/PRJNA778812.

## Ethics Statement

The animal study was reviewed and approved by the Animal Ethics Committee of the Lanzhou Veterinary Research Institute, Chinese Academy of Agricultural Sciences.

## Author Contributions

QN, ZL, and HY conceived and designed the study. HS and DW analyzed RNA-Seq data. HS and QN wrote the manuscript. YRZ performed Q-PCR and ELISA experiments. JY, JF, ZZ, YW, SG, and YLZ participated sample collection and RNA extraction. JY, ZT, JL and GG revised the manuscript. All authors contributed to the article and approved the submitted version.

## Funding

This study was financially supported by the National Natural Science Foundation of China (Grant Nos. 32072830); Gansu Provincial Major Project for Science and Technology Development (Grant Nos. 20ZD7NA006); State Key Laboratory of Veterinary Etiological Biology, Lanzhou Veterinary Research Institute, Chinese Academy of Agricultural Sciences (Grant Nos. SKLVEB2020CGPY02); Basic scientific research business expenses budget incremental project, Chinese Academy of Agricultural Sciences, Lanzhou Veterinary Research Institute (Grant Nos. 1610312021002).

## Conflict of Interest

The authors declare that the research was conducted in the absence of any commercial or financial relationships that could be construed as a potential conflict of interest.

## Publisher’s Note

All claims expressed in this article are solely those of the authors and do not necessarily represent those of their affiliated organizations, or those of the publisher, the editors and the reviewers. Any product that may be evaluated in this article, or claim that may be made by its manufacturer, is not guaranteed or endorsed by the publisher.
