# Supplementary material for: Transcriptome Profiling Reveals Features of Immune Response and Metabolism of Acutely Infected, Dead and Asymptomatic Infection of African Swine Fever Virus in Pigs
Source: Front Immunol. 2021 Dec 15;12:808545. doi: 10.3389/fimmu.2021.808545 (PMC8714921; doi:10.3389/fimmu.2021.808545)
Supplement: Supplementary file 1 [file DataSheet_1.pdf]

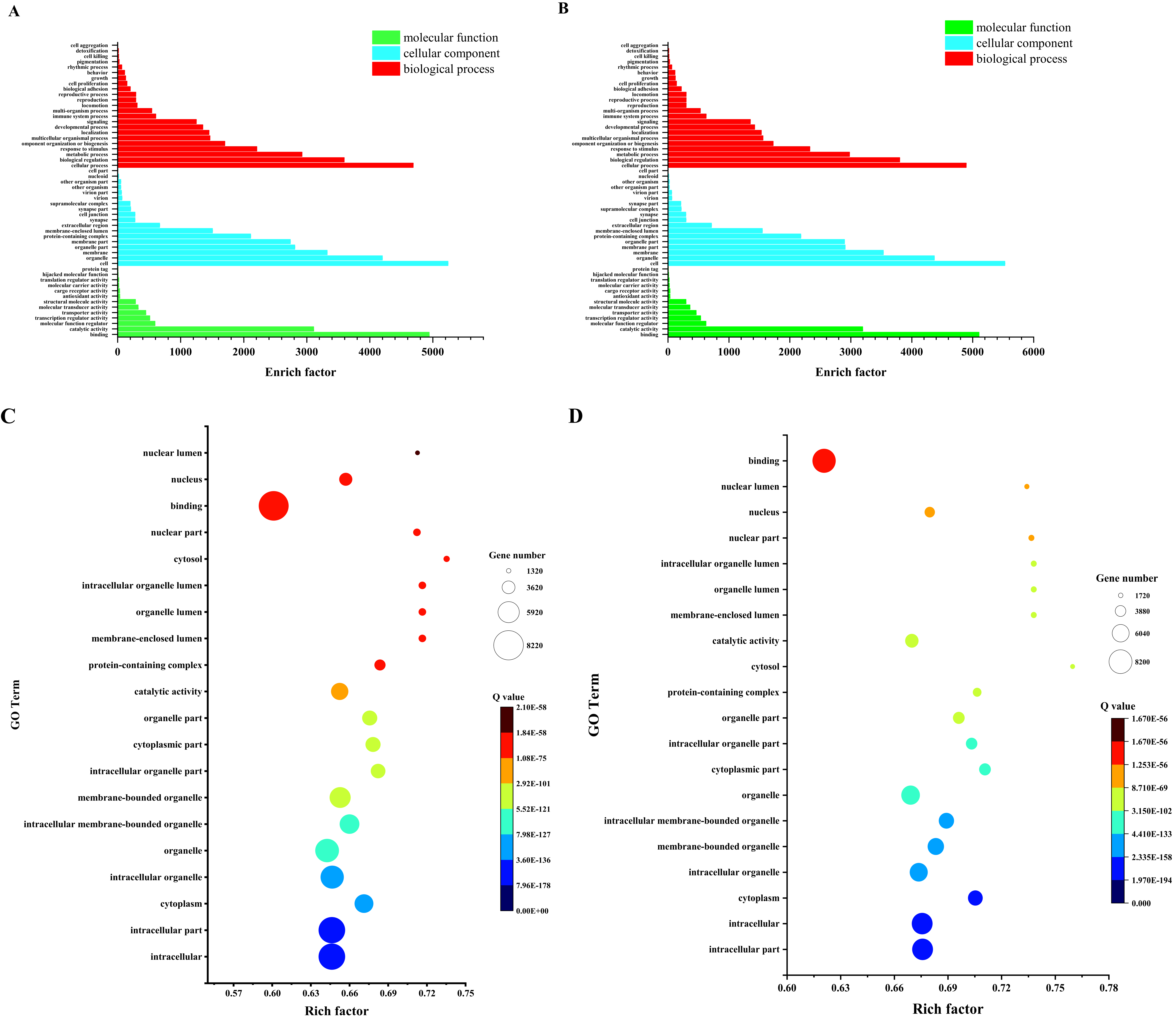

Supplementary Figure S1. GO analysis and enrichment of genes identified in each group. (A) GO analysis of healthy pigs vs. acutely infected, dead pigs. (B) GO analysis of healthy pigs vs. cohabiting asymptomatic pigs. (C) Bubble diagram of GO enrichment in healthy pigs vs. acutely infected, dead pigs. (D) Bubble diagram of GO enrichment in healthy pigs vs. cohabiting asymptomatic pigs.
