# Supplementary material for: Transcriptome Profiling Reveals Features of Immune Response and Metabolism of Acutely Infected, Dead and Asymptomatic Infection of African Swine Fever Virus in Pigs
Source: Front Immunol. 2021 Dec 15;12:808545. doi: 10.3389/fimmu.2021.808545 (PMC8714921; doi:10.3389/fimmu.2021.808545)
Supplement: Supplementary file 2 [file DataSheet_2.pdf]

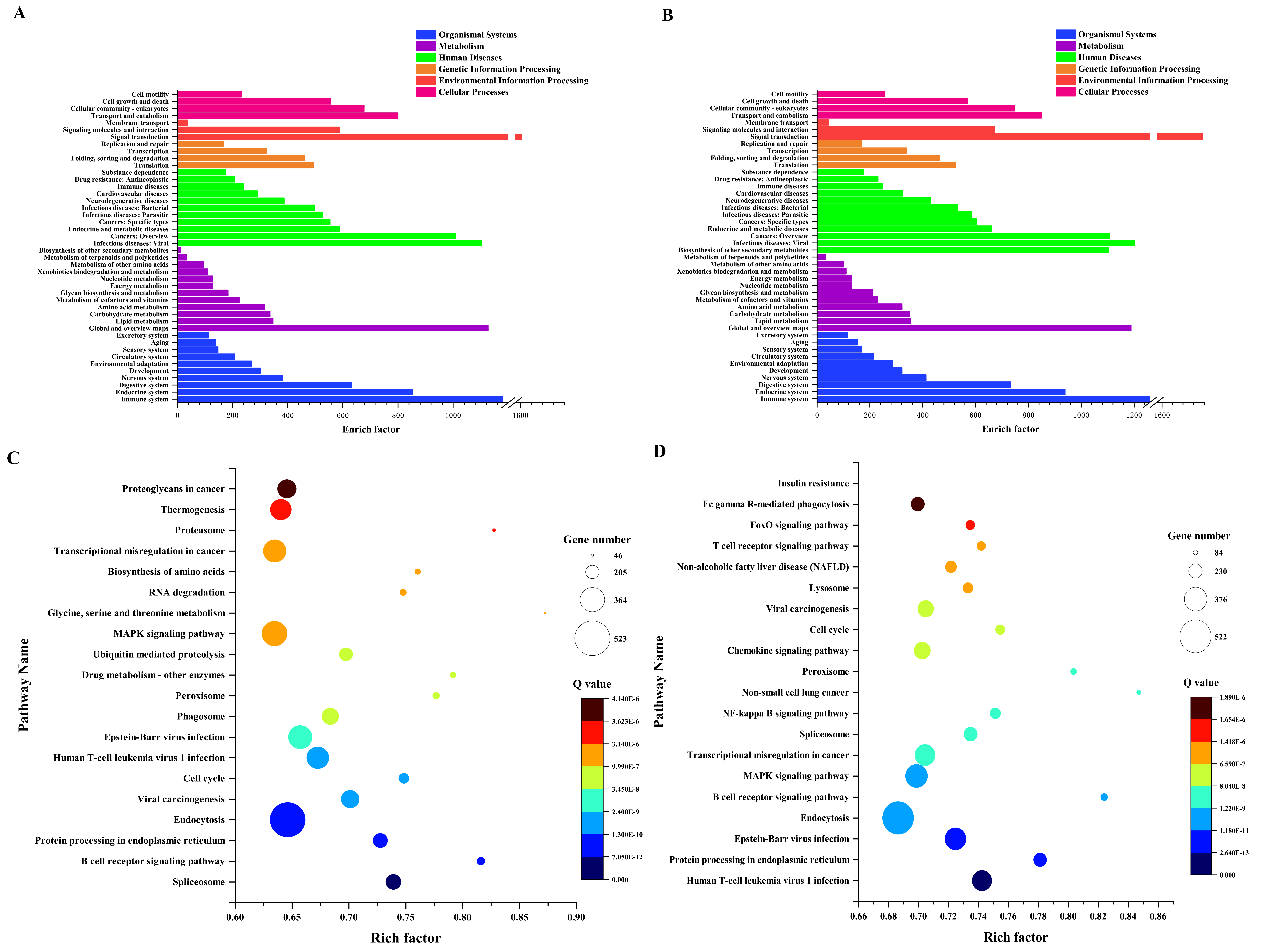

Supplementary Figure S2. KEGG analysis and enrichment of genes identified in each group. (A) KEGG analysis of healthy pigs vs. acutely infected, dead pigs. (B) KEGG analysis of healthy pigs vs. cohabiting asymptomatic pigs. (C) Bubble diagram of KEGG enrichment in healthy pigs vs. acutely infected, dead pigs. (D) Bubble diagram of KEGG enrichment in healthy pigs vs. cohabiting asymptomatic pigs.
